# Role of *Mycobacterium avium* catalase-peroxidase (KatG) in the pathogenesis of MAC disease in HIV patients

**DOI:** 10.1186/1471-2334-12-S1-P15

**Published:** 2012-05-04

**Authors:** Romica Latawa, Ajay Wanchu, Indu Verma

**Affiliations:** 1 Department of Biochemistry, Postgraduate Institute of Medical Education and Research, Chandigarh, India; 2 Department of Internal Medicine, Postgraduate Institute of Medical Education and Research, Chandigarh, India

## Background

The mycobacterial catalase-peroxidases protect it from the reactive oxidative metabolites and allow its survival within the host phagocytes. We compared the virulence of *Mycobacterium avium* complex (MAC) clinical isolates with varying catalase activity recovered from the blood of HIV patients in terms of invasiveness and intracellular multiplicity in host cells. The catalase activity in MAC isolates was also analysed in context to CD4 counts and clinical presentation of mycobacterial disease in HIV patients.

## Methods

Catalase activity of KatG protein of 51 mycobacterial isolates from HIV patients was determined. MAC isolate with maximum catalase activity (KatG-max) was compared to isolate having minimum activity (KatG-min) for adherence, intracellular replication and katG mRNA expression by ZN staining, colony forming units (CFU) enumeration and RT-PCR respectively in A549 and HT29 cell lines.

## Results

Catalase activity of mycobacterial isolates was found to be inversely related to CD4 counts and unrelated to the clinical presentation of mycobacterial disease in HIV patients. The intracellular replication of KatG-max isolate was found to be 2 fold higher than KatG-min at 3^rd^ day of infection (doi) [p<0.001], whereas, it was comparable at 1^st^ doi. CFU enumeration results correlated well with the levels of katGm RNA expression.

## Conclusion

The MAC isolates having maximum catalase activity and increased katGm RNA expression was favoured for its survival and replication in the host cells. High levels of catalase activity in isolates from HIV patients with low CD4 counts suggest an important role of KatG in the establishment and progression of disseminated MAC disease.

